# Unmet health needs and discrimination by healthcare providers among an Indigenous population in Toronto, Canada

**DOI:** 10.17269/s41997-019-00242-z

**Published:** 2019-08-21

**Authors:** George Tjensvoll Kitching, Michelle Firestone, Berit Schei, Sara Wolfe, Cheryllee Bourgeois, Patricia O’Campo, Michael Rotondi, Rosane Nisenbaum, Raglan Maddox, Janet Smylie

**Affiliations:** 1 Department of Public Health and General Practice, NTNU, Trondheim, Norway; 2 Schulich School of Medicine and Dentistry, Western University, Clinical Skills Building, London, Ontario N6A 5C1 Canada; 3 Centre for Urban Health Solutions, Li Ka Shing Knowledge Institute, St. Michael’s Hospital, Toronto, Ontario Canada; 4 Dalla Lana School of Public Health, University of Toronto, Toronto, Ontario Canada; 5 Seventh Generation Midwives Toronto, Toronto, Ontario Canada; 6 School of Kinesiology and Health Science, York University, Toronto, Ontario Canada; 7 Faculty of Health, University of Canberra, Canberra, ACT Australia

**Keywords:** Canada, Health services, Indigenous, Community-based participatory research, Racism, Discrimination, social, Health services accessibility, Canada, Services de santé pour autochtones, Recherche participative basée sur la communauté, Racisme, Discrimination sociale, Accessibilité des services de santé

## Abstract

**Objectives:**

Inequalities between Indigenous and non-Indigenous peoples in Canada persist. Despite the growth of Indigenous populations in urban settings, information on their health is scarce. The objective of this study is to assess the association between experience of discrimination by healthcare providers and having unmet health needs within the Indigenous population of Toronto.

**Methods:**

The Our Health Counts Toronto (OHCT) database was generated using respondent-driven sampling (RDS) to recruit 917 self-identified Indigenous adults within Toronto for a comprehensive health assessment survey. This cross-sectional study draws on information from 836 OHCT participants with responses to all study variables. Odds ratios and 95% confidence intervals were estimated to examine the relationship between lifetime experience of discrimination by a healthcare provider and having an unmet health need in the 12 months prior to the study. Stratified analysis was conducted to understand how information on access to primary care and socio-demographic factors influenced this relationship.

**Results:**

The RDS-adjusted prevalence of discrimination by a healthcare provider was 28.5% (95% CI 20.4–36.5) and of unmet health needs was 27.3% (95% CI 19.1–35.5). Discrimination by a healthcare provider was positively associated with unmet health needs (OR 3.1, 95% CI 1.3–7.3).

**Conclusion:**

This analysis provides new evidence linking discrimination in healthcare settings to disparities in healthcare access among urban Indigenous people, reinforcing existing recommendations regarding Indigenous cultural safety training for healthcare providers. Our study further demonstrates Our Health Counts methodologies, which employ robust community partnerships and RDS to address gaps in health information for urban Indigenous populations.

## Résumé

**Objectifs:**

Des inégalités subsistent au Canada entre les peuples autochtones et non autochtones. Malgré la croissance des populations autochtones en milieu urbain, les informations sur leur santé sont rares. Nous avons voulu évaluer les associations entre les expériences de discrimination par des dispensateurs de soins de santé et la présence de besoins de santé non comblés au sein de la population autochtone de Toronto.

**Méthode:**

Nous avons utilisé la base de données « Our Health Counts Toronto » (OHCT) pour recruter par échantillonnage en fonction des répondants (EFR) 917 adultes de Toronto s’identifiant comme étant Métis, Inuits ou membres des Premières Nations pour répondre à un questionnaire d’évaluation de santé exhaustif. Pour cette étude transversale, nous avons utilisé les données de 836 participants de l’OHCT ayant fourni des réponses à toutes les variables de l’étude. Nous avons estimé des rapports de cotes et des intervalles de confiance de 95 % pour examiner la relation entre l’expérience de discrimination par une dispensatrice ou un dispensateur de soins de santé au cours de la vie et la présence d’un besoin de santé non comblé au cours des 12 mois antérieurs à l’étude. Nous avons procédé à une analyse stratifiée pour comprendre l’influence des données sur l’accès aux soins primaires et les facteurs sociodémographiques sur cette relation.

**Résultats:**

La prévalence de la discrimination par une dispensatrice ou un dispensateur de soins de santé, ajustée selon l’EFR, était de 28,5 % (IC de 95 % 20,4–36,5), et celle des besoins de santé non comblés était de 27,3 % (IC de 95 % 19,1–35,5). La discrimination par une dispensatrice ou un dispensateur de soins de santé était positivement associée à des besoins de santé non comblés (RC 3,1, IC de 95 % 1,3–7,3).

**Conclusion:**

Cette analyse apporte de nouvelles preuves de l’existence d’un lien entre la discrimination dans les milieux de soins et les disparités d’accès aux soins de santé dans les populations autochtones en milieu urbain, ce qui justifie les recommandations existantes de former les dispensateurs de soins de santé à la sécurisation culturelle. L’étude fait aussi la démonstration de la méthode Our Health Counts, qui fait appel à des partenariats associatifs robustes et à l’EFR pour combler les lacunes dans les informations sur la santé des populations autochtones en milieu urbain.

## Introduction

Indigenous peoples of Canada experience enormous health disparities compared with the general population of Canada, stemming from current and historical health inequities (Adelson 2005; Allan and Smylie 2015; Smylie et al. 2011; Truth and Reconciliation Commission of Canada 2015; Smylie and Adomako 2009). The health status of Indigenous peoples in Canada must be understood within the context of current and historical colonial policies implemented by the Canadian government and other colonial institutions, from the loss of land and autonomy, to the creation of the reserves systems, the historical removal of Indigenous children into residential schools, and the current removal of Indigenous children by the child welfare system (Adelson 2005; Allan and Smylie 2015; Smylie et al. 2011; Truth and Reconciliation Commission of Canada 2015; Smylie and Adomako 2009). Despite these health inequities, critical gaps remain in our understanding of Indigenous health in Canada (Adelson 2005; Allan and Smylie 2015; Smylie et al. 2011). Health data sources systematically fail to fully cover Indigenous populations for two reasons: inconsistent, inconclusive, and unreliable documentation of Indigenous identity in primary datasets; and sampling methods that systematically exclude significant population segments (non-Status First Nations, Métis, Indigenous populations in urban settings, those who are housing-insecure or homeless, among others) (Smylie and Firestone 2015; Rotondi et al. 2017). We do not have good representative data to understand and address health and social needs for the growing proportion of Indigenous peoples who live in urban centres (Statistics Canada 2008). Research that is conducted in partnership with communities and adheres to Indigenous research principles, such as OCAP® (Ownership, Control, Access and Possession), is considered critical to generating valid and methodologically rigorous data to address these knowledge gaps (Smylie et al. 2012a).

Growing evidence points to the negative health outcomes resulting from abuse and discrimination in health settings (Reader and Gillespie 2013). Beliefs and attitudes of healthcare providers contribute to procedural neglect (care behaviour that falls short of the standards that constitute good care) and caring neglect (care behaviour that leads to the belief that healthcare staff “do not care”), both of which negatively impact patient health and the care they receive (Reader and Gillespie 2013). A 2015 review on the impacts of racism and colonialism on Indigenous health in Canada expanded the discussion, identifying epistemic racism (positioning knowledge of one racialized group as superior to another and passing judgement on what constitutes knowledge) and internalized racism (acceptance and internalization of an ideology of racial inferiority) (Allan and Smylie 2015). Studies among Indigenous peoples in urban settings have shown a high prevalence of experiences of discrimination through negative behaviours such as insults and unfair treatment, including in healthcare settings (Senese and Wilson 2013; Environics Institute 2010; Browne et al. 2011). Qualitative studies of experiences of discrimination indicated many Indigenous patients were attuned to negative body language and non-verbal communication from healthcare providers (Browne et al. 2011). Past experiences of discrimination by health professionals grow into assumptions regarding the potential for future discrimination by other health professionals which may create barriers to seeking healthcare.

Legislative loopholes and jurisdictional ambiguities between the provincial/territorial governments responsible for general healthcare provision and federal levels of government providing health services to some First Nations and Inuit communities result in confusion regarding where and how Indigenous peoples can access healthcare (Lavoie 2013). For many Indigenous people, contact with the healthcare system for primary care for non-urgent health needs is forced to occur at emergency departments (EDs), though many choose to avoid health services altogether (Allan and Smylie 2015; Lavoie 2013). The 2011 Our Health Counts (OHC) Hamilton comprehensive health study of the urban First Nations of the city of Hamilton, Ontario, conducted in partnership with the community-based De dwa da dehs nye>s Aboriginal Health Access Centre, found overrepresentation of First Nations peoples in the ED for acute and non-acute illness (Smylie et al. 2011). The prevalence of at least one visit over the past 2 years was 52% for urban First Nations compared with 22% for the general Hamilton population (Smylie et al. 2011). EDs are not designed to respond fully and effectively to the complex health and social issues that require longitudinal and interdisciplinary care, and false perceptions of ED misuse for primary care may perpetuate racist assumptions within healthcare providers (Allan and Smylie 2015; Browne et al. 2011). Understanding the prevalence of self-reported unmet health needs provides insight into the ability of the health system to function effectively.

The primary objective of this paper is to examine the key association between ever experiencing discrimination by a healthcare provider and having an unmet health need in the last 12 months. Secondary objectives include examination of the impact of specific social determinants of health, including access to a regular healthcare provider and socio-demographic factors, on the key association under study.

## Methods

### Community-based participatory research partnership

*Our Health Counts Toronto (OHCT): Developing a Population Based Urban Aboriginal Cohort to Assess and Enhance Individual, Family, and Community Health and Wellbeing* is a comprehensive Indigenous health study in an urban setting, conducted using Indigenous community-based participatory research principles. OHCT was designed and implemented in partnership between the Well Living House action research centre within the Centre for Urban Health Solutions (C-UHS) at St. Michael’s Hospital in Toronto, and Seventh Generation Midwives Toronto (SGMT), a midwifery practice focused on serving the urban Indigenous community. While the data are stored on secure servers at the Well Living House, the Indigenous community partner SGMT owns and maintains control over what data are released, to whom and for what purpose. There was also a project reference group comprised of over 20 local and regional Indigenous and allied health and social service organizations that met quarterly to help guide the research process. These rightsholders were involved throughout the study process, including survey design and question development, data analyses, interpretation, and sharing of the results to facilitate careful consideration of underlying local Indigenous community processes and protocols. For example, the reference group identified priority survey domains, tested identified questions for validity within the local context, and supported piloting the survey. Building on existing OHC studies and existing survey tools, this process provided rigorous feedback throughout the development process to ensure applicability to the local community. Results were also disseminated to study participants by a community event and report. The aforementioned engagement processes align with the ethical guidelines used for the research conducted by the Royal Commission on Aboriginal Peoples (RCAP) and the principles of OCAP®, which assist to ensure Indigenous control over Indigenous research data (OCAP® is a registered trademark of the First Nations Information Governance Centre (FNIGC) 1998; Canada, Royal Commission on Aboriginal Peoples 1993). Ethics approval was provided by the Well Living House Counsel of Grandparents and SGMT. The OHCT study also received ethics clearance from the St. Michael’s Hospital Research Ethics Board (REB# 14-083c).

### Respondent-driven sampling

The OHCT data were collected using respondent-driven sampling (RDS) methodology to expand our understanding of Indigenous health and impact of discrimination by healthcare providers in urban settings. As initially detailed by Heckathorn, RDS was developed to collect data from “hidden” or marginalized populations, populations for which no adequate sampling frame exists (i.e., simple random sampling is inadequate) and where identifying as a member of the population may carry real or perceived repercussions (Heckathorn 1997; Heckathorn 2002). RDS distinguishes itself from typical traditional chain referral sampling methods, such as convenience and snowball sampling. RDS examines the social network of respondents in a recruitment chain and develops post-sampling weights related to the probability of recruitment (Heckathorn 1997; Heckathorn 2002). Heckathorn has shown that as recruitment progresses, a sample selected develops a random composition distinct from the characteristics of the initial respondents or “seeds” and start locations (Heckathorn 1997). With sufficient recruitment waves, the characteristics of the sample recruited reach an equilibrium and approximate a random sample of the general population (Heckathorn 1997; Heckathorn 2002). The equilibrium state, or convergence, is typically reached after 4–6 waves of participants (Gile and Handcock 2010; Salganik and Heckathorn 2004). RDS methodology has the potential to generate accurate population-level estimates and associations, and has been utilized to examine other Indigenous populations in urban settings, including First Nations in Hamilton and Inuit in Ottawa (Smylie et al. 2011; Rotondi et al. 2017; Firestone et al. 2014; Rotondi 2013; Smylie et al. 2012b).

As detailed in Rotondi et al. (2017), the target sample size was 1200 Indigenous adults (aged 15+ years) derived from a sample size calculation with an assumed RDS design effect of 2.5 to provide appropriate power for descriptive and comparative measures (Firestone et al. 2014; Salganik 2006). This target was refined based on recruitment patterns and lengths of RDS chains as the study progressed.

Inclusion criteria for the adult study were self-identification as Indigenous, ≥ 15 years old, and residency in the City of Toronto and/or recipient of health and/or social services in the City of Toronto. Initial RDS “seed” participants (*n* = 20) were selected from diverse Indigenous identity, socio-economic, educational, occupational, geographic, gender, and age backgrounds (Johnston and Sabin 2010). Each participant was able to recruit up to five additional participants until the sample size was attained. The incentives utilized within the OHCT were developed through discussion and dialogue with SGMT and agencies and organizations in the reference group and are aligned with incentives used for similar research (Smylie et al. 2011). Incentives were set at $20 CAD for completion of the 90-min survey and $10 CAD for every successful new participant a respondent recruited, up to a maximum of five.

Following standard RDS procedures, social network information was collected from each participant. Study coupons were uniquely numbered to keep track of who recruited whom. Social network size was determined by answering the following question: “Approximately how many Aboriginal people do you know (i.e., by name and who know you by name) who currently live, work or use health and social services in Toronto?”

All data in the OHCT survey were collected between March 2015 and March 2016. Interviews were conducted onsite at three well-known organizations in accessible areas of the city. Several home visits were made for interviews with less mobile individuals.

Following recruitment and informed consent, one-on-one interviews were used to complete a respectful health survey lasting approximately 90 min. All interviewers were Indigenous community members, which contributed to creating culturally safe spaces for the interviews.

The decision was made to stop recruitment in March 2016 as the sample of Indigenous adults (*N* = 940) was deemed adequate based on the estimated sampling variability for our primary study outcomes. The longest recruitment chain was 19 waves.

The “respectful health assessment survey tool” utilized in this study was built upon the tools developed for the OHC Hamilton, which incorporated domains of relevance to the Indigenous community identified through concept mapping, and was modified for the OHCT through discussions with community partners (Smylie et al. 2011). Unmet health needs, a validated metric of health equity, attempts to gauge whether health services are adequately meeting health needs (Statistics Canada 2016). Reasons for self-reported unmet health needs may be related to issues of availability, accessibility, and acceptability (Sibley and Glazier 2009). Self-reported unmet health needs were measured by asking “In the previous 12 months, was there a time you felt you needed healthcare services but did not receive them?” Lifetime experiences of discrimination by a healthcare provider were determined by asking “Have you ever been treated unfairly (e.g., treated differently, kept waiting) by a health professional (e.g., doctor, nurse, etc.) because you are Aboriginal?”

### Statistical analysis

Existing univariate and multivariable analysis methods for examining associations are not directly transferable to RDS samples because random sampling assumptions are not satisfied. Bivariate associations between exposure to discrimination by a healthcare provider and the outcome of an unmet health need were explored using unadjusted odds ratios. Following this, stratified odds ratios were calculated for each social determinant of health, including access to a regular healthcare provider and socio-demographic factors (gender, age, education level, employment status, food security, mobility in the past 12 months, and income level).

RDS weights were calculated, using the RDS-II weighting estimator developed by Volz and Heckathorn, along with wave number, for each eligible participant through the RDS Analyst program, powered by the statistical package R (Volz and Heckathorn 2008; Handcock et al. 2014). Prevalence estimates and unstratified and stratified odds ratios for the associations of interest were calculated with 95% confidence intervals in SAS® University Edition (SAS Institute Inc. SAS® University Edition for OS X 2016).

## Results

**Fig. 1:**
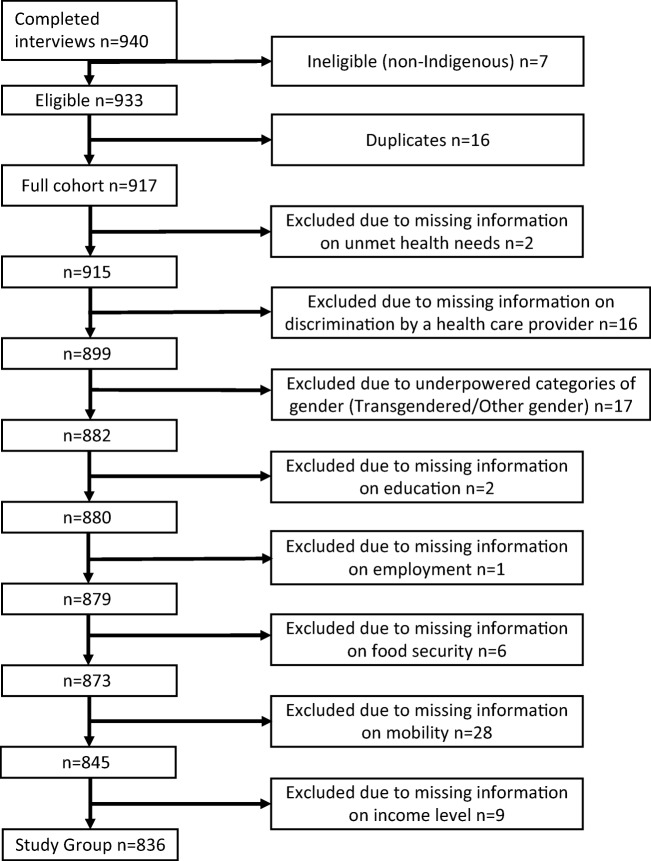
Flow chart of inclusion in the study sample

A total of 940 interviews were conducted, with a final number of survey respondents eligible for inclusion in the study sample at 836 (Fig. 1). Interviews not included in the analyses were removed because of ineligibility, duplication, missing information from key questions, or small sample size categories. One non-Indigenous respondent with Indigenous children was kept for the RDS calculations. This was to ensure recruitment chains were not disrupted. However, they were excluded from the final sample. Seventeen participants were removed due to small cell counts in the subsequent analysis of their gender identities (transgendered/other gender). We established that this exclusion did not bias our findings. The prevalence estimate of exposure to discrimination by a healthcare provider among transgender/other gender participants (28.0%, 95% CI 0.0–62.3) was close to that of the study sample. The discrimination exposures of transgendered people in the healthcare system are unique and need further study with a sufficiently powered sample. The RDS-weighted population prevalence estimates of the sample of 836 approximate the RDS-weighted population prevalence estimates derived from the full cohort of 917 respondents (those eligible but excluded due to missing information from key questions or small sample size categories), with significant overlap of 95% confidence intervals. Socio-demographic frequencies for the sample, with RDS-weighted population prevalence estimates, are presented in Table 1. RDS design effects for variables of interest, such as unmet health needs, were estimated as approximately 4. After RDS weight calculation and prevalence adjustment, the majority of the Indigenous population were First Nations (85.7%, 95% CI 79.7–91.7). The Indigenous population was also young, with 30.2% (95% CI 21.9–38.4) between the ages of 15 and 29 years old. The prevalence of self-reported unmet health needs in the past 12 months in the Indigenous population was 27.3% (95% CI 19.1–35.5). Experience of discrimination by a healthcare provider in this Indigenous population in an urban setting was reported by 28.5% (95% CI 20.4–36.5). Access to a regular healthcare provider, such as a physician or nurse practitioner, was reported by 63.1% (95% CI 54.6–71.6) of the Indigenous population.

**Table 1:** Characteristics of the study sample

	Sample frequency *n* = 836 (%)	RDS-adjusted prevalence estimate (95% CI)
Gender		
Female	444 (53.1)	49.3 (41.0, 57.6)
Male	392 (46.9)	50.7 (42.4, 59.1)
Indigenous identity		
First Nations	735 (87.9)	85.7 (79.7, 91.7)
Inuit	11 (1.3)	0.4 (0.1, 0.7)
Métis	80 (9.6)	13.2 (7.3, 19.1)
First Nations and Métis	X^1^	0.4 (0.0, 1.0)
Other	X^1^	0.3 (0.0, 0.8)
Age		
15–29	174 (20.8)	30.2 (21.9, 38.4)
30–39	181 (21.6)	22.0 (14.7, 29.4)
40–49	186 (22.2)	17.8 (13.1, 22.5)
50+	295 (35.3)	30.0 (22.5, 37.5)
Education		
Did not complete high school	357 (42.7)	50.5 (42.2, 58.8)
Completed high school or more	479 (57.3)	49.5 (41.2, 57.8)
Employment		
Employed	261 (31.2)	17.8 (13.4, 22.2)
Not in labour force	140 (16.8)	20.0 (12.7, 27.3)
Unemployed	435 (52.0)	62.2 (54.4, 70.0)
Food security		
Always enough food	177 (21.2)	20.1 (13.0, 27.1)
Enough but not of desired foods	434 (51.9)	55.0 (46.9, 63.2)
Sometimes not enough	150 (17.9)	17.9 (12.9, 22.9)
Often not enough	75 (9.0)	7.1 (4.0, 10.1)
Mobility in the past year		
0	508 (60.8)	48.2 (39.9, 56.4)
1	177 (21.2)	29.7 (22.1, 37.3)
2 or more moves	151 (18.1)	22.1 (14.0, 30.2)
Income^2^		
Below low-income cutoff	662 (79.2)	87.4 (84.0, 90.8)
Above low-income cutoff	174 (20.8)	12.6 (9.2, 16.0)
Unmet health needs in the past 12 months		
Yes	197 (23.6)	27.3 (19.1, 35.5)
No	639 (76.4)	72.7 (64.5, 81.0)
Access to a regular healthcare provider		
Yes	572 (68.4)	63.1 (54.6, 71.6)
No	264 (31.6)	36.9 (28.4, 45.4)
Discrimination by a healthcare provider		
Yes	263 (31.5)	28.5 (20.4, 36.5)
No	573 (68.5)	71.5 (63.5, 79.6)

^1^X indicates suppressed cell due to low counts

^2^Low-income cutoff (LICO) based on 2014 Statistics Canada LICO cutoffs

Unstratified and stratified odds ratios and 95% confidence intervals are presented in Table 2. Odds ratios were calculated for the Indigenous population as a whole as small cell counts affected the ability to analyze Métis and Inuit subgroups. In the unstratified analysis, exposure to discrimination was associated with significantly higher odds of unmet health needs, crude OR 3.1 (95% CI 1.3–7.3), when compared with those who had not been discriminated against by a healthcare provider. When the association between discrimination by a healthcare provider and unmet health needs was stratified by specific social determinants of health under investigation, additional potentially modifying factors are identified. For example, among Indigenous people who did not have access to a regular healthcare provider, those who had been discriminated against by a healthcare provider had over five times higher odds (OR 5.2, 95% CI 1.2–22.3) of reporting unmet health needs. Beyond access to a regular healthcare provider, as detailed in Table 2, factors that appear to modify the association between discrimination by a healthcare provider and unmet health needs included gender, age, employment status, food security, and income.

**Table 2:** The association between exposure to discrimination by a healthcare provider and having unmet health needs, with stratified analysis of this association among respondents within each stratum. Significant RDS-adjusted associations are italicized

	No discrimination by a healthcare provider		Discrimination by a healthcare provider		
All (*n* = 836)	Unmet health needs (*n*)	No unmet health needs (*n*)	Unmet health needs (*n*)	No unmet health needs (*n*)	RDS-adjusted OR estimate (95% CI)
Unstratified analysis of the total sample:	97	476	100	163	*3.1 (1.3, 7.3)*
Stratified analysis of respondents in each stratum:					
Access to a regular HCP					
Yes	61	328	64	119	2.3 (0.8, 6.6)
No	36	148	36	44	*5.2 (1.2, 22.3)*
Gender					
Female	51	225	68	100	1.5 (0.6, 4.3)
Male	46	251	32	63	*5.9 (1.6, 21.2)*
Age					
15–29	22	106	16	30	1.8 (0.4, 8.8)
30–39	23	99	25	34	2.8 (0.5, 15.8)
40–49	21	99	29	37	2.0 (0.6, 6.6)
50+	31	172	30	62	*7.0 (1.3, 37.2)*
Education					
Did not complete high school	36	208	38	75	*3.9 (1.1, 13.3)*
Completed high school or more	61	268	62	88	*3.4 (1.1, 10.6)*
Employment					
Employed	24	160	26	51	0.7 (0.3, 2.0)
Not in labour force	21	74	20	25	2.5 (0.4, 15.7)
Unemployed	52	242	54	87	*4.3 (1.4, 13.5)*
Food security					
Always enough food	14	115	7	41	X^1^
Enough but not of desired foods	49	254	55	76	*5.2 (1.6, 16.8)*
Sometimes not enough	23	73	22	32	3.5 (0.8, 14.2)
Often not enough	11	34	16	14	0.7 (0.1, 5.2)
Mobility in the past year					
0	54	303	57	94	1.8 (0.8, 3.9)
1	23	100	17	37	3.4 (0.9, 13.1)
2 or more	20	73	26	32	2.1 (0.3, 15.9)
Income					
Below LICO	86	370	78	128	*3.2 (1.2, 8.5)*
Above LICO	11	106	22	35	1.5 (0.5, 4.9)

^1^X indicates RDS-adjusted OR calculation included at least one cell less than 15

## Discussion

The findings of this study identify a strong crude relationship (OR 3.1, 95% CI 1.3–7.3) between experiences of discrimination by a healthcare provider and unmet health needs. This supports the community and rightsholder understanding that experiences of discrimination by healthcare providers are a determinant of unmet health needs. Further, this contributes evidence to the significant negative impact of discrimination in the healthcare setting (Environics Institute 2010; Paradies 2006; Benjamins and Whitman 2014). The strength of this relationship is consistent with past findings regarding discrimination by healthcare providers and unmet health needs. A 2014 study found discrimination by healthcare providers to increase odds (OR 2.5, 95% CI 1.6–3.9) of unmet health needs among minority groups in the USA (Benjamins and Whitman 2014). A 2006 systematic review of empirical research on self-reported racism and health found the strongest and most consistent association quantified was between racism and negative mental health (Paradies 2006). Associations between racism and negative physical health were also identified within the review (Paradies 2006).

The pervasive experiences of discrimination by healthcare providers, with 28.5% of the Indigenous population of Toronto having ever experienced discrimination, must be addressed. The need for Indigenous cultural safety training in medical and nursing schools in Canada, particularly “skills-based training in intercultural competency, conflict resolution, human rights, and anti-racism” has been identified within the 94 Calls to Action of the Truth and Reconciliation Commission of Canada (TRC) *(Truth and Reconciliation Commission of Canada*^2015^*)*. Cultural safety training programs, such as the San’yas Indigenous Cultural Safety Training program developed in British Columbia, which has been adapted for Ontario (provincial Indigenous Cultural Safety program) and Manitoba, provide an opportunity for healthcare providers to learn about their internalized biases that cause them to make discriminatory assumptions leading to modified care (Browne et al. 2011; Hole et al. 2015). While cultural safety training has potential to address implicit bias, existing evaluative literature is sparse (Churchill et al. 2017). This is a key priority alongside ensuring that programs are mandated within healthcare organizations and government to support structural changes to help address systemic and institutional discrimination (Truth and Reconciliation Commission of Canada 2015; Churchill et al. 2017).

The results also confirm the community and rightsholder understanding that primary care is commonly inaccessible to the Indigenous population of Toronto, with only 63.1% of the population having a regular healthcare provider, such as a doctor or a nurse practitioner. When the crude relationship between discrimination and unmet healthcare needs was stratified by those without access to healthcare provider, the strength of the association increased from OR 3.1 (95% CI 1.3–7.3) to OR 5.2 (95% CI 1.2–22.3). This finding provides empirical evidence for the increased impact that not having a regular healthcare provider can have on the relationship between discrimination by a healthcare provider and unmet health needs. While access to a regular healthcare provider does not guarantee all healthcare needs are met, a shortage of culturally safe health service providers coupled with an increase in short-term interactions with healthcare providers at walk-in clinics and EDs may increase the potential for discrimination by healthcare providers and affect continuity of care for Indigenous patients (Browne et al. 2011).

Indigenous peoples in urban settings may be at a higher risk of not receiving healthcare when they felt they needed it, due to increased risk of discrimination when living within a dense concentration of non-Indigenous people. Systemic issues in health service provision for Indigenous peoples who have moved from a home community or reserve may also contribute to this risk (Lavoie 2013). The estimate that 27.3% of the Indigenous population in Toronto has self-reported unmet health needs in the past 12 months is significantly higher in comparison with the national estimate of 16.2% of Indigenous peoples (those who self-identified as First Nations, Inuit, or Métis and were living outside of a reserve or Indigenous community) from the 2014 Canadian Community Household Survey (CCHS) (Statistics Canada 2014). The CCHS estimated 11.2% of non-Indigenous people nationally had a self-reported unmet health need (Statistics Canada 2014). The provincial rate of unmet health needs in Ontario, including both Indigenous and non-Indigenous people, was estimated at 10.3% (Statistics Canada 2014). An important aspect of unmet health needs is the lack of adequate Indigenous specific services, including services that are perceived as intrinsic to the Indigenous community. As research on prenatal and infant-toddler health promotion programs has demonstrated, and in alignment with TRC Calls to Action, services and programs that are intrinsic to, and involve Indigenous community investment and ownership can trigger community activation and participation, such as an Indigenous midwifery practice (e.g., SGMT) or housing and support for Indigenous men (e.g., Na-Me-Res or Native Men’s Residence) (Truth and Reconciliation Commission of Canada 2015; Smylie et al. 2016).

### Strengths and limitations of this study

Strengths of this study include the Indigenous community governance and investment in this project from preconception to implementation and dissemination. This close relationship to the community has allowed for the successful gathering of a large sample of a hard-to-reach population recruited using RDS. One-on-one interviews were used to complete the survey. All interviewers were Indigenous community members, which contributed to creating culturally safe spaces for the interviews. Interview respondents were generous with their time and the stories they related, which speaks to their desire to share and the safe space successfully created by the interviewers.

This study is not without limitations. For example, RDS methodology requires large sample sizes because design effects are large. The analysis was often underpowered, manifesting in wide confidence intervals, which in many cases could not exclude the possibility of no significant association, despite large effect size point estimates. The inability to use multivariable analysis methods on this RDS sample necessitated using a sequential stratification approach to including relevant covariates, with examination of one socio-demographic covariate at a time.

Another limitation arises from attempting to recruit specific Indigenous subpopulations, including Métis and Inuit within a larger inclusive Indigenous sample. Some groups may be undersampled if they have fewer social network ties to other groups within the larger sample (Firestone et al. 2014). In the OHCT, there is potential evidence of this undersampling of Métis in that they represented only 9.6% of the total sample and 13.6% of the RDS-adjusted population, well below the expected amount of over 30% found in the 2006 Census (Statistics Canada 2008). However, examining the upper bound of the 95% CI for this proportion, a population prevalence of up to 19.1% Métis cannot be ruled out, which may not be inconsistent given the many limitations of the Census and our comparison with results from a previous decade (Rotondi et al. 2017). Nonetheless, the distribution of Métis within the recruitment chains indicates little presence of separate Métis social networks, i.e., individuals were well distributed throughout the chains rather than clustered. It is possible that despite the rigorous preparation and dissemination of information regarding the study, only a subpopulation of Métis was recruited and there may be a substantive additional subpopulation of Métis who were excluded from sampling. The social isolation of Métis from each other and from the larger Indigenous community could be explained by current and historic policies, including exclusion of Métis from Indigenous services (structured to preferentially serve First Nations people with Status), disruptions of Métis kin-networks, and the historic vilification of Métis in Ontario which resulted in it becoming unsafe for Métis to reveal their identity (Lavoie 2013).

Self-identification or perceived racism is the benchmark method for research in discrimination; however, under-reporting is common in many populations experiencing racism (Allan and Smylie 2015). Racism is entrenched in everyday lives, with an estimated 53.1% (95% CI 44.8–61.4) of the Indigenous population of Toronto ever experiencing discrimination because of their Indigenous identity. Healthcare providers may also provide differential treatment without either the patient or the healthcare provider being aware of differential treatment, through implicit bias (Allan and Smylie 2015). Similarly, individuals experience their own health differently and it is probable that generations of racist and colonial experiences have made it less likely that Indigenous people will indicate having an unmet health need, as a manifestation of internalized racism (Allan and Smylie 2015). The question regarding unmet health needs is also complicated by differing concepts of health between the Indigenous concepts of holistic well-being and the Western biomedical tradition (Allan and Smylie 2015).

## Conclusions

The comprehensive population-based health assessment database for First Nations, Inuit, and Métis living in Toronto produced by the OHCT study provides a unique opportunity to identify and address health inequalities. The research contributes to the growing evidence base of the negative health impacts associated with discrimination in healthcare settings among Indigenous peoples in urban settings, a significant relationship strengthened by not having a regular healthcare provider. This evidence reinforces calls for healthcare providers to receive cultural safety training to address implicit bias. These OHCT findings were made possible through robust community partnership and RDS methodology which were integral to, and have contributed to filling key information gaps on determinants of healthcare access for the Indigenous population living in Toronto.
